# Triggering antibacterial activity of a common plant by biosorption of selected heavy metals

**DOI:** 10.1007/s00775-024-02045-1

**Published:** 2024-04-08

**Authors:** Mária Kováčová, Halyna Bodnár Yankovych, Adrian Augustyniak, Mariano Casas-Luna, Michaela Remešová, Lenka Findoráková, Martin Stahorský, Ladislav Čelko, Matej Baláž

**Affiliations:** 1 Institute of Geotechnics, Slovak Academy of Sciences, Watsonova 45, 040 01 Košice, Slovakia; 2 Chair of Building Materials and Construction Chemistry, Technische Universität Berlin, Gustav-Meyer-Allee 25, 13355 Berlin, Germany; 3 Faculty of Chemical Technology and Engineering, The West Pomeranian University of Technology in Szczecin, Piastów Avenue 42, 71 065 Szczecin, Poland; 4 Institute of Biology, University of Szczecin, ul. Wąska 13, 71-415 Szczecin, Poland; 5 Central European Institute of Technology, Brno University of Technology, Purkyňova 656/123, 612 00 Brno, Czech Republic; 6 Faculty of Mathematics and Physics, Charles University, Ke Karlovu 3, 121 16 Prague 2, Czech Republic

**Keywords:** Biosorption, Lead, Copper, *Thymus serpyllum* L. plant, Antibacterial action

## Abstract

The presented study proposes an efficient utilization of a common *Thymus serpyllum* L. (wild thyme) plant as a highly potent biosorbent of Cu(II) and Pb(II) ions and the efficient interaction of the copper-laden plant with two opportunistic bacteria. Apart from biochars that are commonly used for adsorption, here we report the direct use of native plant, which is potentially interesting also for soil remediation. The highest adsorption capacity for Cu(II) and Pb(II) ions (*q*_e_ = 12.66 and 53.13 mg g^−1^, respectively) was achieved after 10 and 30 min of adsorption, respectively. Moreover, the Cu-laden plant was shown to be an efficient antibacterial agent against the bacteria *Escherichia coli* and *Staphylococcus aureus*, the results being slightly better in the former case. Such an activity is enabled only via the interaction of the adsorbed ions effectively distributed within the biological matrix of the plant with bacterial cells. Thus, the sustainable resource can be used both for the treatment of wastewater and, after an effective embedment of metal ions, for the fight against microbes.

**Graphical abstract:**

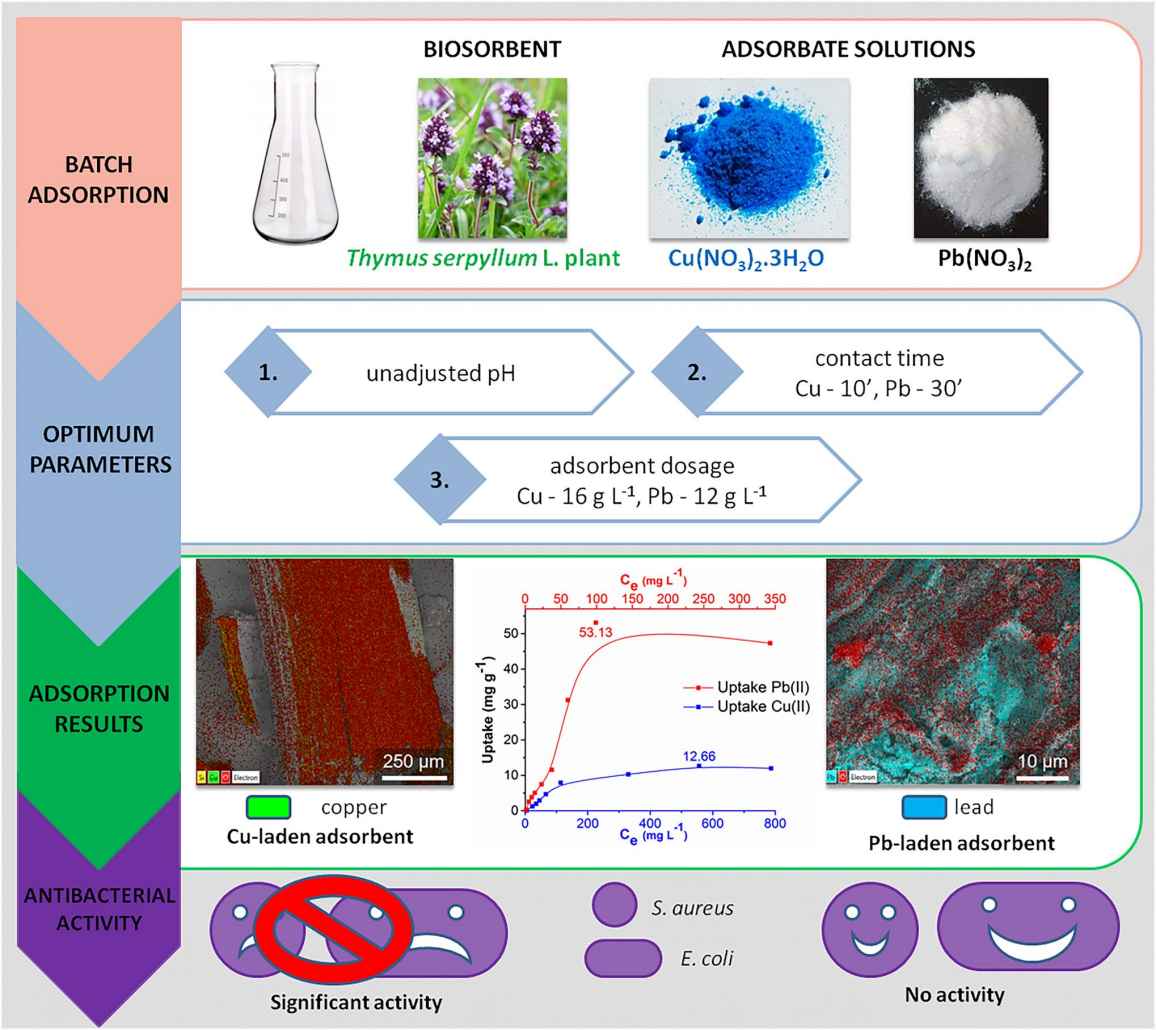

**Supplementary Information:**

The online version contains supplementary material available at 10.1007/s00775-024-02045-1.

## Introduction

The contamination of drinking water and wastewaters by heavy metal ions is a very important environmental issue, as the intoxication with those substances can cause various health problems, with the central and peripheral nervous system, gastrointestinal, cardiovascular, haematopoietic and renal systems being the most susceptible to damage [1]. Copper and lead belong to the most common heavy metal contaminants [2]. Lead is a good example of a typical heavy metal with a number of harmful effects when accumulated in the human body even at low concentrations [3]. The principal targets for lead toxicity are the central and peripheral nervous systems, but bones and kidney are affected as well [2]. Moreover, lead inhibits the biosynthesis of haem and accelerates the destruction of erythrocytes which causes anaemia [4]. Copper is generally considered less harmful than lead, although the Cu(II) ions represent the most toxic form of copper for aquatic species [5]. The presence of Cu(II) ions in drinking water can cause diarrhoea, vomiting, abdominal pain, nausea, chronic disorders and kidney damage [6]. When ingested at high concentrations, copper promotes oxidation and may be a risk factor for cancer [7]. Cu(II) ions are extremely dangerous for people with Wilson disease, which is a genetic disorder that causes the accumulation of copper in different tissues and, if left untreated, may damage the liver, central nervous system and in the worst case cause death [8]. According to the DIRECTIVE (EU) 2020/2184, the maximum allowed concentration in water intended for human consumption for Cu(II) ions is 2.0 mg L^−1^. In relation to Pb(II) ions, the parametric value of 5.0 μg L^−1^ shall be met, at the latest, by 12 January 2036. The parametric value for lead until that date shall be 10.0 μg L^−1^ [9].

Among the many available methods for water remediation (e.g. reverse osmosis, ion exchange, electrodialysis, ultrafiltration), the process of adsorption and specifically biosorption has an inevitable place and seems to be preferable because of low operation costs [10, 11]. The search for sustainable materials as adsorbents is one of the most developing areas of research these days [12, 13]. Nature-based materials such as bacteria, algae, fungi, plants, biopolymers, the skin of animals and fruits or even natural waste materials have attracted increasing attention as biosorbents for heavy metals owing to their renewable, biodegradable and environmentally friendly properties [14–17].

Biosorbents derived from plants include a wide range of materials such as visible parts of plants (shoots, bark and foliage), roots and seasonal parts (blossom, fruit, seeds, stones, hulls, husks etc.) [18–20]. This is closely related to phytoremediation, where plants are used to purify both soil and water [21]. Plant-derived materials consist of biopolymers, mostly various forms of lignocellulose (lignin, cellulose, hemicellulose, etc.) and tannins that contain specific functional groups in their structure (e.g. hydroxyl, carboxyl, carbonyl, thiol, amine and others) allowing binding of chemical compounds. The mechanism of biosorption is very complex and consists of a number of physical and chemical processes such as adsorption (physical, chemisorption), ion exchange, complexation or microprecipitation. Each biosorbent has its own characteristics such as a chemical structure or porosity that affects its sorption capacity and reactivity under given conditions. The physical and chemical parameters (pH, temperature, biosorbent dosage, etc.) also influence the effectiveness of biosorption [22].

*Thymus serpyllum* L. (wild thyme) is an aromatic flowering plant belonging to the *Lamiaceae* family, which is widely used for medicinal as well as food seasoning purposes [23]. *Thymus serpyllum* L. contains high levels of essential oils and polyphenolic compounds, which are either phenolic acids (e.g. chlorogenic, caffeic and rosmarinic acids) or flavonoids (e.g. luteolin and apigenin glucuronide) that are responsible for high radical scavenger potential and anti-inflammatory, anticancer, antioxidant and antibacterial properties [24, 25]. It is also known to be useful for phytoremediation as it is able to accumulate some heavy metal ions from the contaminated soils [26]. Its ability to adsorb antimony ions was also reported [27].

However, although if the adsorption (including biosorption) is successful, the question what to do with the metal-laden adsorbent arises. There are more options as well summarized in [28], including their utilization of post-adsorption products as antimicrobial agents, e.g. silver- [29], zinc- or copper-containing [30, 31] sorbents have already been used in this way.

The aim of the present paper was to investigate the adsorption of Cu(II) and Pb(II) ions from their model solutions on the native *Thymus serpyllum* L. (wild thyme) plant and investigate the potential application of the heavy metal-laden adsorbent as an antibacterial agent. It was hypothesized that copper(II) and lead(II) ions would be efficiently adsorbed on to the plant due to the presence of many functional groups available for interaction with them, and subsequently the presence of adsorbed heavy metal ions in the plant matrix would trigger antibacterial activity due to their efficient interaction with bacteria.

## Materials and methods

### Materials

*Thymus serpyllum* L. (wild thyme) plant (SER) was purchased from a local company, Agrokarpaty, s.r.o., Slovakia, which cultivates medicinal plants. The plant was milled in a kitchen mixer and sieved to reach a particle size ≤ 1 mm. Copper nitrate trihydrate (Cu(NO_3_)_2_·3H_2_O), lead nitrate (Pb(NO_3_)_2_), cadmium nitrate tetrahydrate (Cd(NO_3_)_2_·4H_2_O), zinc nitrate hexahydrate (Zn(NO_3_)_2_·6H_2_O), iron nitrate nonahydrate (Fe(NO_3_)_3_·9H_2_O), aluminium chloride hexahydrate (AlCl_3_·6H_2_O) and cobalt chloride hexahydrate (CoCl_2_·6H_2_O) were used as chemicals without further purification for the preparation of model solutions. All chemicals were purchased from Merck KGaA, Germany, and ITES Vranov, s.r.o., Slovakia, and were of analytical grade.

### Methods

#### Adsorption studies

Adsorption experiments were performed at least twice at laboratory temperature in a batch mode in Erlenmeyer flasks on the orbital shaker. The information about the preliminary adsorption experiment and the initial experiments with Cu(II) and Pb(II) ion adsorption can be found in Table S1.

##### Preliminary experiments

At first, the adsorption potential of SER plant was investigated in a model solution prepared from a corresponding mass of chemically pure Cd(NO_3_)_2_·4H_2_O, Zn(NO_3_)_2_·6H_2_O, Cu(NO_3_)_2_·3H_2_O, Pb(NO_3_)_2_, Fe(NO_3_)_3_·9H_2_O, AlCl_3_·6H_2_O and CoCl_2_·6H_2_O dissolved in distilled water to obtain a concentration 20 mg L^−1^ for each element in the final solution. Such a low concentration was selected because 100% adsorption of metal ions from the solution was expected. The mass of adsorbent (SER sample) was 0.12 g, the volume of the model solution was 0.06 L and the contact time was 2 h.

Based on the experiment mentioned above, we chose Cu(II) and Pb(II) for the consecutive experiments to scrutinize the adsorption potential of SER. As a first step, we studied the adsorption of Cu(II) and Pb(II) at three different concentrations (*c* = 20, 200 and 1000 mg L^−1^). Higher concentrations were chosen to investigate selective adsorption to study competitive adsorption. The adsorption was performed in the same way as had already been mentioned, but the contact time was 5, 10, 15, 30, 45, 60, 90 and 120 min. The mass of adsorbent was reduced to 0.06 g and the volume of copper/lead nitrate solution was 0.03 L, which means the adsorbent dosage remained unchanged as in the case of the experiment with the solution containing many ions described above.

##### Adsorbent dosage

Thereafter, subsequent experiments were conducted to determine the optimal adsorption parameters. The experimental conditions were maintained as a mass of adsorbent 0.06 g and volume of copper/lead nitrate solution 0.03 L with concentration 200 mg L^−1^. During the experiment for defining the best adsorbent dosage, the concentrations 0.4, 0.8, 1, 2, 3, 4, 8, 12, 16, 20 and 24 g L^−1^ were examined.

##### pH investigation

The dependence of the adsorption capacity on pH was analysed in the pH range from 1 to 5 for Cu(II) ions and from 1 to 6 for Pb(II) ions. The pH values of Cu(NO_3_)_2_·3H_2_O and Pb(NO_3_)_2_ solutions were adjusted by adding an appropriate amount of 0.1 M NaOH or 0.1 M HNO_3_ solution.

##### Kinetic studies

To study the adsorption kinetics of Cu(II) and Pb(II) ions, SER sample with concentration 2 g L^−1^ (0.06 g in 0.03 L) and metal-containing model solutions with concentration 200 mg L^−1^ were stirred for 1, 3, 5, 7, 10, 15, 30 and 60 min. To find the optimal kinetic model for the studied process, the experimental data were fitted to Lagergren’s pseudo-first [32] and Ho’s pseudo-second-order [33] kinetic models.

##### Isotherms modelling

The adsorption isotherm investigations were performed in the concentration range 10–1000 mg L^−1^ at the optimal adsorption conditions determined by previous experiments for Cu(II) and Pb(II) ions, respectively. The adsorption isotherm data were processed by the linearized forms of equations for Langmuir and Freundlich isotherm models and the appropriate parameters were calculated [34–36].

##### Competitive adsorption

To investigate the influence of metal ion coexistence on the adsorption, the binary solution of both metals was prepared. The binary solution contained Cu(NO_3_)_2_·3H_2_O and Pb(NO_3_)_2_ with the concentration 200 mg L^−1^ for each heavy metal (to achieve competitiveness). The mass of the adsorbent was 0.48 g, the volume of binary solution was 0.03 L, the agitation time was 30 min and the pH was natural.

##### Estimation of the adsorption mechanism

To determine whether the ion exchange process was involved in the adsorption of Cu(II) and Pb(II) ions, we measured the content of alkali (Na(I), K(I)) and alkaline Earth (Ca(II), Mg(II)) cations released into the aqueous solution from pure SER plant as described in [37] and the total content of released cations was expressed in mEq g^−1^. Afterwards, the adsorption of Cu(II) and Pb(II) ions on the SER biosorbent after washing and drying was performed under the optimal conditions identified by the previous experiments. Namely, for the adsorption of Cu(II) ions, 0.48 g SER and 10 min contact time were used, and for the adsorption of Pb(II) ions 0.36 g SER and 30 min contact time were applied. In both cases, the ionic concentration of 750 mg L^−1^ was used, as the maximum adsorption capacity for both target metals was reached at this point.

The adsorption capacity (or uptake) at equilibrium *q*_e_ or at time *t* *q*_t_ (mg g^−1^) of the adsorbent and the removal percentage of the adsorbed heavy metals were calculated according to Eqs. 1 and 2 described in [38, 39]:

1 $$q_{{\text{e/t}} } = \frac{{\left( {C_0 - C_{\text{e/t}} } \right)V}}{m},$$

2 $$R = \frac{{C_{{\text{o}} } - C_{\text{t}} }}{{C_{\text{o}} }} \times 100\% ,$$

where *C*_0_ (mg L^−1^) is the initial concentration, *C*_e_ or *C*_t_ (mg L^−1^) is the concentration in equilibrium or at time *t*, respectively, *V* (L) is the volume of the adsorbate solution and *m* (g) is the mass.

#### Characterization methods

X-ray diffraction (XRD) patterns of the samples after adsorption were obtained using a D8 Advance diffractometer (Bruker, Billerica, MA, USA) with CuKα radiation of 0.15407 nm wavelength at 40 kV accelerating voltage and 40 mA electric current. The samples were scanned over a 15°–70° range of diffraction angle (2*θ*).

The value of the specific surface area of the initial plant was obtained by using a NOVA 1200e Surface Area and Pore Size Analyzer (Quantachrome Instruments, FL, USA) using the Brunauer–Emmet–Teller (BET) methodology. The samples were degassed in vacuum for 2 h at laboratory temperature before the measurement.

The grain size analysis was performed using a particle size laser diffraction analyser Mastersizer 2000E (Malvern Panalytical, Malvern, UK) in the dry mode. The sample was measured three times.

The concentration of copper(II), lead(II), cadmium(II), zinc(II), iron(III), aluminium(III) and cobalt(II) ions in the initial and residual adsorption solutions as well as the concentration of calcium(II), magnesium(II), potassium(I) and sodium(I) ions in the filtrate after washing of pure SER plant were determined by atomic absorption spectrometry (AAS) using an atomic absorption spectrometer SPECTRAA L40/FS (Varian, Crawley, UK).

The morphology of the samples and elemental distribution were studied by a scanning electron microscope (SEM) Lyra3 (Tescan, Czech Republic) equipped with an energy-dispersive X-ray spectroscopy (EDX) unit X-Max 50 (Oxford Instruments, UK). The SEM/EDX examination was carried out in back-scattered electron (BSE) and secondary electron (SE) modes using an acceleration beam voltage of 10 keV. The EDX mapping spectra were analysed using AZtec software (Oxford Instruments NanoAnalysis, UK). Prior to SEM/EDX examination, each dry SER sample was placed on double-sided adhesive carbon tape and coated with a 20 nm-thick carbon layer using a sputter coater Leica EM ACE600 (Leica Microsystems, Germany).

Simultaneous TG/DTG and DTA analysis of the studied compounds were carried out using STA 449 Jupiter thermal analyser (Netzsch, Germany). A 30 mg of sample was placed in an Al_2_O_3_ crucible and heated from 25 °C up to 1300 °C at a linear heating rate 5 °C min^–1^ in synthetic air (N_2_ 80%, O_2_ 20%) combined with an argon atmosphere.

FT-IR spectra were recorded using a Tensor 29 infrared spectrometer (Bruker, Germany) using the ATR method in the range 4000–650 cm^−1^.

The X-ray photoelectron spectroscopy (XPS) of the pure SER plant and after Cu and Pb adsorption was performed in XPS—Kratos Axis Supra apparatus (Manchester, UK) working with a monochromatic Al–Kα radiation at an emission current of 15 mA. Wide and high-resolution spectra were acquired with a pass energy of 80 eV and 20 eV, respectively. The spectra were calibrated setting the C1*s* emission at 284.7 eV. The deconvolution and fitting of the interested elements, i.e. Cu2*p*, Pb4*f*, C1*s*, and O1*s*, were carried out using the CasaXPS software (version 2.3.22) by applying a Spine-Shirley background and a Gaussian/Lorentzian (70/30) fitting for the individual high-resolution spectra peaks.

The zeta potential (ζ-potential) measurements were determined using a Zetasizer Nano ZS (Malvern, UK). Prior to measurements, 20 mg of each sample was sonicated in 10 mL of 10 mM NaCl solution for 10 min. NaCl solution was used to maintain a minimum level of conductivity. ζ-potential values were obtained by applying the Smoluchowski equation built into the Malvern Zetasizer software. The measurements were repeated three times for each sample.

#### Antibacterial activity assessment

For the evaluation of the antibacterial activity of the heavy metal-laden adsorbents, respiration studies on two bacterial models—*Escherichia coli* ATCC^®^ 25922™ and *Staphylococcus aureus* ATCC^®^ 33591™ were performed. The microorganisms were kept frozen at − 20 °C in Trypticase soy broth (TSB, Biomaxima, Lublin, Poland) medium with 20% v/v glycerol. Before each test, microorganisms were revived on Trypticase soy agar (TSA, Biomaxima, Lublin, Poland) medium and incubated at 37 °C.

The samples were prepared by transferring its dry form to an Eppendorf-type tube (1.5 mL). Afterwards, each tube was filled with bacteria suspension. The concentration used in the analysis was 2 mg mL^−1^. For comparison, also the antibacterial activity of the solutions of pure metal nitrates was investigated. Bacteria for the experiments were suspended in PBS from an overnight culture in TSB and diluted to reach approximately 1 × 10^9^ CFU mL^−1^. Controls for the experiments included a positive control without the tested samples, positive control containing 40 µg mL^−1^ of gentamicin and negative control without bacteria.

The antibacterial activity was tested in the prepared suspensions in a 4-h toxicity test. Samples were kept dark for 4 h and their respiration was monitored for an additional 4 h in resazurin assay. After the first incubation, 10% (v/v) of resazurin (1 mg mL^−1^, Merck, Darmstadt, Germany) was added to the cultures and incubated at 37 °C with fluorescence measurements (*λ*_ex_ = 520 nm, *λ*_em_ = 590 nm) recorded every 10 min.

## Results and discussion

### Brief characterization of the initial plant powder

The initial plant powder obtained after the size reduction in the kitchen mixer and subsequent sieving under 1 mm was characterized by SEM, particle size distribution and specific surface area. The elongated micrometre-scale particles detected by SEM (Fig. S1) were the residues of the successful cutting of the initial plant. It is clear that also some particles with high aspect ratio above 1 mm in length were present in the powder, as they passed the sieve with width well below 1 mm. Some irregularities on the surface were detected upon higher magnifications (Fig. S1b, c). The particle size distribution (Fig. S2) confirmed that the powder was well below 1 mm in size, showing unimodal distribution with the maximum around 250 µm. There are clearly also finer particles as the shoulder peak to the left of the maximum reaches zero value at around 1 µm. The specific surface area value was 1.8 m^2^ g^−1^, which was very low compared to that of biochars that are generally used for biosorption [40].

### Preliminary adsorption experiments

#### The experiment with model solution

According to the preliminary experiment with a model solution containing a combination of various ions (see Table S1), *Thymus serpyllum* L. plant (SER) showed the best adsorption potential towards Fe(III), Cu(II) and Pb(II) ions, as the concentration of these three ions decreased markedly in comparison with the other four tested ones. In agreement with the obtained results, we chose to focus on the adsorption of Cu(II) and Pb(II) in further experiments. We did not study the adsorption of Fe(III) ions further because at pH > 4.0, Fe(III) ions precipitate in the form of Fe(OH)_3_ and therefore very acidic conditions are required for their adsorption [41, 42] and some structures of the plant adsorbent would most probably decompose under such conditions. Moreover, Cu(II) and Pb(II) ions are more toxic and more dangerous in lower concentrations than Fe(III) ions.

#### Adsorption performance at different concentrations

The adsorption capacity values (*q*_t_) for the adsorption of Cu(II) and Pb(II) ions at three different concentrations (*c* = 20, 200 and 1000 mg L^−1^) showed the most consistent results at *c* = 200 mg L^−1^ (see Fig. S3); thus, we worked with this concentration in the subsequent experiments.

### Determination of the optimal adsorption parameters

#### The effect of pH

**Fig. 1:**
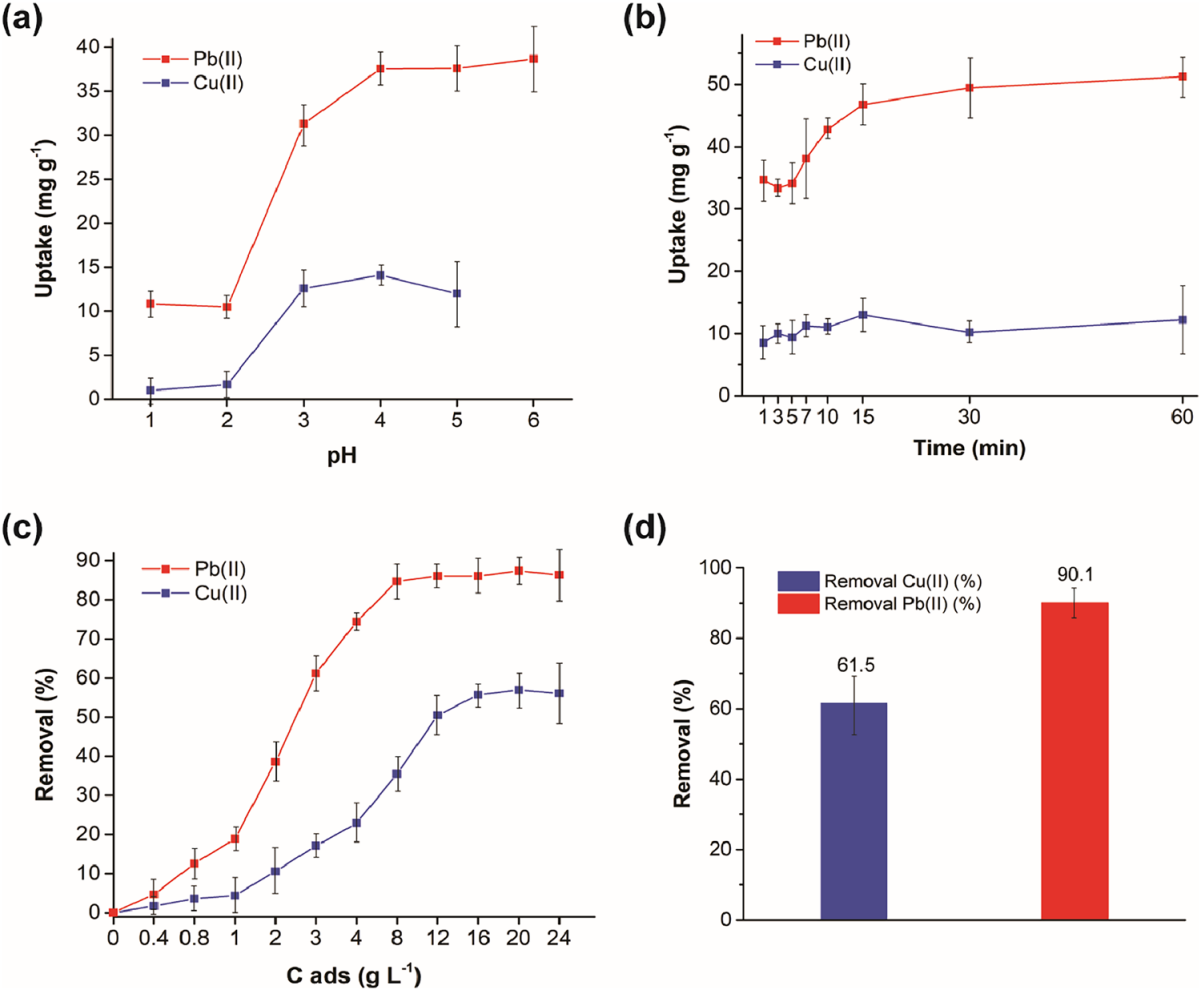
Effect of the different variables on the adsorption of Cu(II) and Pb(II) ions onto *Thymus serpyllum* biosorbent: **a** pH; **b** contact time; **c** adsorbent dosage; **d** adsorption from the binary metal solution

pH is one of the most important parameters affecting the adsorption of metal ions, because the surface charge of an adsorbent can be modified by changing the pH of the solution and partly due to the fact that hydrogen ions are strong competing sorbates [43]. To discover the influence of this parameter on the adsorption of Cu(II) and Pb(II) ions, metal solutions with different pH values ranging 1–6 were prepared due to the precipitation of metals in the form of insoluble substances at higher pH values. At very acidic pH (1 and 2), the uptake of Cu(II) and Pb(II) ions was very low, and with increasing pH the uptake of both ions increased (Fig. 1a). The best results for Cu(II) ions were achieved at pH = 4, and for Pb(II) ions the uptake in the pH range from 4 to 6 was approximately the same. Considering that the natural unadjusted pH of both nitrate solutions was in the optimal pH range, we continued with following experiments at natural pH. The pH of distilled water containing the SER plant after 24 h of staying was 6.6. The study by Littera et al. [27] on the adsorption of Sb(III) ions on the same plant revealed that Sb(III) removal was the highest in the pH range 3.3–4.6; however, the authors selected pH 5.6 for further experiments.

#### The effect of contact time

To study the appropriate contact time for the adsorption of Cu(II) and Pb(II) ions, kinetic experiments were performed (Fig. 1b). The initial uptake after the first minute was quite high for both ions (*q*_t_ = 8.5 mg g^−1^ and 34.7 mg g^−1^ for Cu(II) and Pb(II) ions, respectively) and later slowed down, which was more prominent in case of Cu(II) adsorption. The uptake of Cu(II) ions was constant after 10 min (*q*_t_ = 11 mg g^−1^) and in the case of Pb(II) ions after 30 min (*q*_t_ = 49.4 mg g^−1^); therefore, these contact times were selected in further experiments.

#### The effect of adsorbent dosage

The dosage of adsorbent is another important parameter affecting the adsorption ability of the studied biosorbent. Figure 1c displays that we examined 11 different SER concentrations (starting from 0.4 g L^−1^ up to 24 g L^−1^) in our experiments. The removal rate for both heavy metal ions increased gradually and the plateau was reached at 16 g L^−1^ with 55.7% removal for Cu(II) ions and at 12 g L^−1^ with 86.1% removal for Pb(II) ions. Further increase in the adsorbent dosage did not result in higher removal of the studied pollutants. The adsorbent dosage 16 g L^−1^ was found to be optimal for the adsorption of Sb(III) ions when using the same plant in the paper by Littera et al. [27].

#### The binary system adsorption

To explore the combined adsorption onto SER biosorbent, an adsorption experiment with a binary metal system containing both Cu(II) and Pb(II) ions was performed. The results presented in Fig. 1d show that the removal efficiency was higher for Pb(II) ions and was not affected by the presence of both metal ions in the solution, as the removal 61.5% and 90.1% for Cu(II) and Pb(II) ions, respectively, is very similar to the results presented in Fig. 1c, thus suggesting that different functional groups are involved in the adsorption process of these two ions.

### Adsorption kinetics

To analyse the adsorption kinetics of the studied metals, pseudo-first- and pseudo-second-order equations were applied and the obtained kinetic parameters are summarized in Table 1. According to these results, the adsorption of Cu(II) and Pb(II) ions follows the pseudo-second-order model, as the correlation coefficients are close to 1 (*R*^2^ = 0.9997 and 0.9961, respectively). Littera et al. [27] also achieved a higher correlation coefficient for the pseudo-second-order model during the adsorption of Sb(III) ions. This suggests that the chemisorption of Cu(II) and Pb(II) ions is the rate-determining step of the adsorption process and involves the chemical interaction such as ion exchange or chelation between Cu(II) and Pb(II) ions and polar functional groups on the adsorbent surface [44]. The better fit of the pseudo-second-order model also indicates the existence of two types of active adsorption sites on the adsorbent surface [36].

**Table 1:** Kinetic parameters for adsorption of Cu(II) and Pb(II) ions onto *Thymus serpyllum* L. plant

Metals	Pseudo-first order			Pseudo-second order			
	ln(*q*_e_ − *q*_t_) = ln *q*_e_ − *k*_1_ t			*t*/*q*_t_ = 1/(*k*_2_ *q*_e_^2^) + *t*/*q*_e_			
	*k*_1_	*q*_e, cal_	*R*^2^	*k*_2_	*q*_e, cal_	*R*^2^	*q*_e, exp_
Cu(II)	1.6 × 10^–2^	1.87	5.1 × 10^–2^	0.30	21.98	1.00	12.66
Pb(II)	4.64 × 10^–2^	16.61	0.92	8.93 × 10^–2^	50.51	1.00	53.13

*q*_e, cal_, *q*_e, exp_ (mg g^−1^) the calculated and the experimental adsorption capacity at equilibrium time, respectively, *q*_t_ (mg g^−1^) the adsorption capacity at time *t*, *k*_1_ (min^−1^) and *k*_2_ (g mg^−1^ min^−1^) the kinetic rate constants of the pseudo-first-order and pseudo-second-order models, respectively, *R*^2^ correlation coefficient

### Adsorption isotherms

**Fig. 2:**
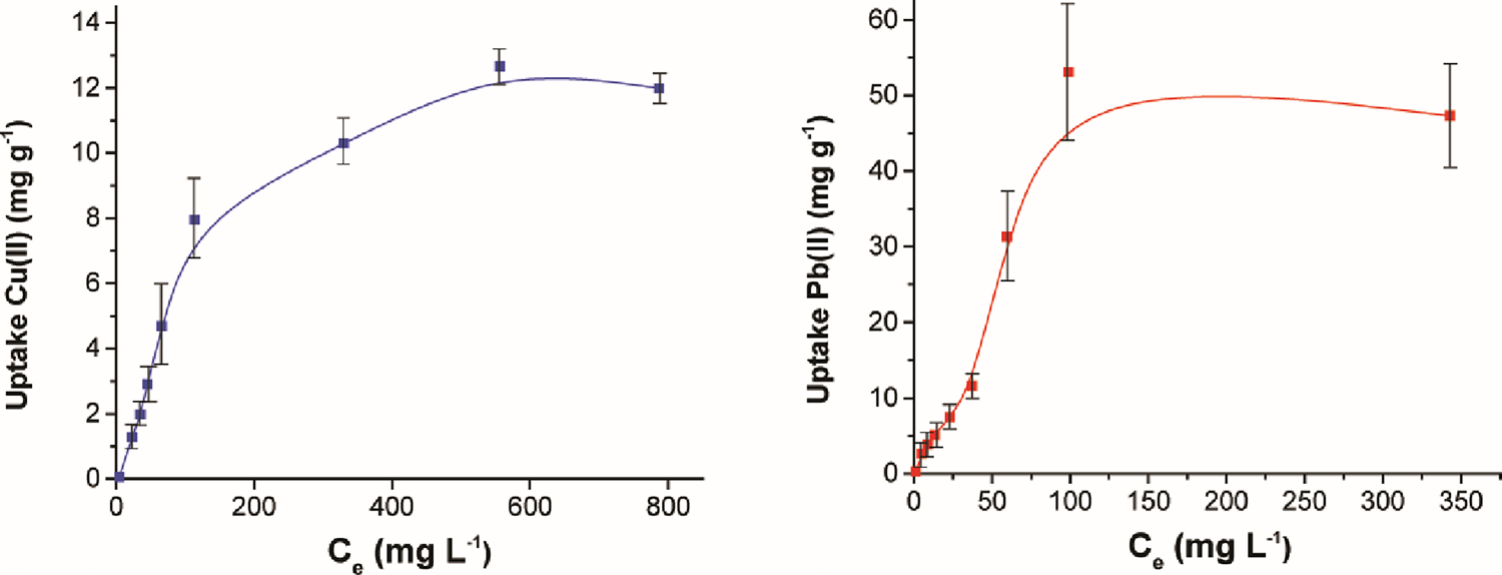
The adsorption isotherms for the adsorption of **a** Cu(II) and **b** Pb(II) ions on *Thymus serpyllum* biosorbent

The experimental adsorption isotherms (i.e. dependences of the amount of adsorbed heavy metal ions in mg per g adsorbent on the equilibrium concentration of the ions) are presented in Fig. 2. The main adsorption characteristics were analysed by fitting the experimental results using two mostly used models (Langmuir and Freundlich isotherm model) and the obtained parameters are summarized in Table 2. The Langmuir equation is valid for the monolayer adsorption onto a surface of the adsorbent with a finite number of identical sites [45]. In our case, the Langmuir model was better suited for the adsorption of Cu(II) ions, which implied that the adsorption of Cu(II) was limited to one molecular layer and the maximum monolayer adsorption capacity—*Q*_m_ was calculated to be 14.6 mg g^−1^. This model was also found to be suitable for the adsorption of Sb(III) ions in [27], and the observed adsorption capacity therein was 8.8 mg g^−1^. In the case of Pb(II) ion adsorption in our study, the value of correlation coefficient was higher for Freundlich isotherm model which suggests that the adsorption occurred on the heterogeneous surface of the adsorbent. The favourability and capacity of the adsorbent/adsorbate system are related to the magnitude of 1/n and the higher fractional values of 1/n suggest that the system has strong adsorption forces [46].

**Table 2:** Adsorption isotherm parameters for Cu(II) and Pb(II) ions adsorption onto *Thymus serpyllum* plant

Metals	Langmuir model				Freundlich model			
	*C*_e_/*q*_e_ = 1/(*K*_L_ × *Q*_m_) + *C*_e_/*Q*_m_				ln *q*_e_ = ln *K*_F_ + (1/*n*) × ln *C*_e_			
	*K*_L_	*Q*_m_	*q*_e exp_	*R*^2^	*K*_F_	1/*n*	*n*	*R*^2^
Cu(II)	7.3 × 10^–3^	14.64	12.66	0.98	8.07	0.81	1.23	0.91
Pb(II)	1.82 × 10^–2^	27.40	53.13	0.86	3.79	1.14	0.88	0.98

*K*_L_ (L mg ^−1^) the Langmuir adsorption equilibrium constant, *Q*_m_ (mg g^−1^) the maximum monolayer adsorption capacity, *q*_e, exp_ (mg g^−1^) the experimental adsorption capacity at equilibrium time, *R*^2^ correlation coeficient, *K*_F_ (L g^−1^) the Freundlich constant indicating the adsorption capacity, *n* (g L^−1^) the Freundlich constant representing the adsorption intensity

According to [47], the difference in selectivity and adsorption capacity of Cu(II) and Pb(II) ions onto an adsorbent can be associated with the physicochemical properties of these two ions such as the differences in ionic radius, electronegativity, hydrated radius and atomic weight.

In Table 3, the performance of our biosorbent is compared with that reported in few other selected studies. Out of seven reports selected for comparison, six of them were devoted to the adsorption of both copper(II) and lead(II) ions, similar to our study.

**Table 3:** Comparison of maximum adsorption capcities of Cu(II) and Pb(II) ions removal for various biosorbents used in native state

Sorbent	Maximum sorption capacity (mg/g)		References
	Cu	Pb	
*Thymus vulgaris* leaves	38.93	–	[48]
*Myrica esculenta* leaves	39.37	43.89	[49]
Water hyacinth leaves	32.47	49.30	[50]
Shiitake stalks	22.7	130.0	[51]
Champion stalks	31.4	87.0	[51]
Fungi grown on corncob	1.77	14.75	[52]
Banana peels	28.56	66.67	[53]
Ginkgo leaves	57.80	117.64	[54]
Peanut shells	9.36	33.33	[54]
Metasequoia leaves	43.85	108.69	[54]
Dried wild thyme powder	12.66	55.13	This work

All the biosorbents mentioned in the table were used in their native state, i.e. no biochar was produced, as usually just drying below 100 °C was applied. The comparison clearly shows that the biosorption ability of wild thyme with regard to copper was poor, whereas that of lead was average. Nevertheless, the plant we used is very common and thus its lower performance could potentially be counter-balanced by its high abundance. Moreover, if we are aiming for potential antibacterial activity, the essential oil of this plant is known to exhibit it [25, 55, 56], which might be of added value in this particular application.

### Possible mechanism of Cu(II) and Pb(II) ion adsorption onto *Thymus serpyllum* L.

The investigation of the adsorption mechanism is a very important issue with the potential to predict and control the adsorption process. Therefore, the available advanced methods such as SEM/EDX, TGA, FT-IR, XPS and ζ-potential measurements were utilized to estimate the removal mechanism during the adsorption of Cu(II) and Pb(II) ions on the SER surface. Previous studies have revealed that the elimination of these contaminants can be realized by different processes, among which the complexation with surface carboxylic groups, the chelation with nitrogen moieties with formation of a coordinate bond between the lone pair of nitrogen and *d*-orbitals of heavy metals and the ion exchange are the most common [57–59].

**Fig. 3:**
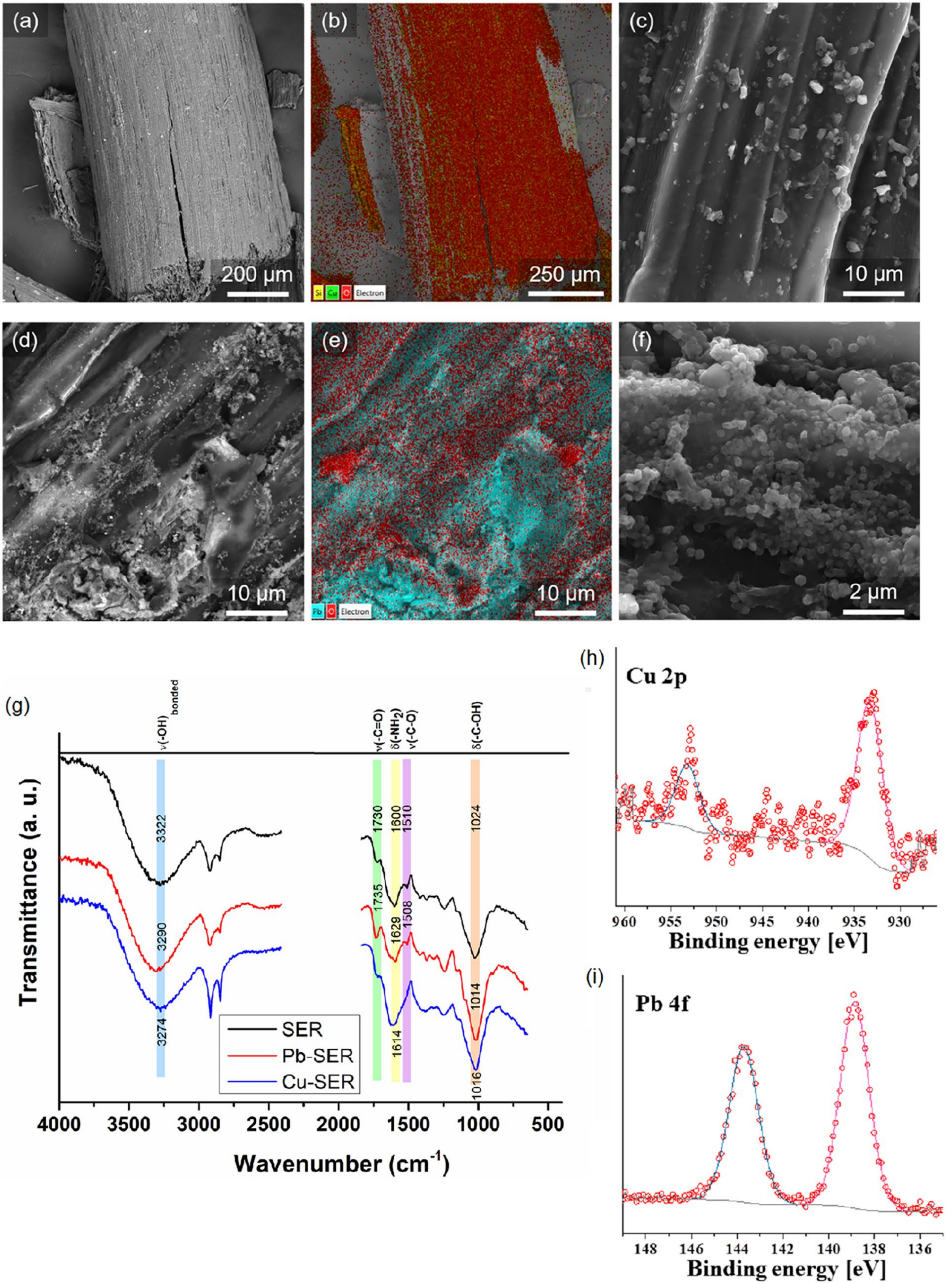
Characterization of metal-laden adsorbent: **a**–**f** SEM micrographs and EDX mapping of *Thymus serpyllum* biosorbent surface after the adsorption of **a**–**c** Cu(II) and **d**–**f** Pb(II) ions; **a**, **d** surface morphology overview (SEM–BSE); **b**, **e** EDX layered map, **c**, **f** surface details (SEM–SE); **g** FT-IR spectra of native *Thymus serpyllum* biosorbent and the samples after the adsorption of Pb(II) (Pb-SER) and Cu(II) (Cu-SER) ions; **h**, **i** high-resolution X-ray photoelectron spectra of **h** Cu-SER (Cu 2*p* spectra) and Pb-SER (Pb 4*f* spectra)

To prove the adsorption process as well as to estimate the distribution of the adsorbed ions on the biosorbent surface, SEM/EDX observations were done. As illustrated in Fig. 3, the adsorbed Cu(II) and Pb(II) ions are evenly distributed on the SER surface in the form of precipitates, as small spheres and particles with indefinite shape for Cu and Pb adsorption, respectively (Fig. 3c, f). The EDS mapping showed the inhomogeneous distribution of Cu and Pb elements (Fig. 3b, e). The amount of lead in the selected region in Fig. 3e is much higher than that of Cu in Fig. 3b.

To determine the nature of the precipitates, the XRD measurements of the metal-laden adsorbents were performed. However, no crystalline phases could be identified on the diffractograms (see Fig. S4a, b). Hence, it can be suggested that the available amount of crystalline phase cannot be detected because of the interfering effect of the amorphous plant matrix (this might be the reason in the case of Cu(II) ions adsorption) or because the precipitates most probably have an amorphous nature (this might be the case of Pb(II) ions adsorption).

To better understand the processes present during the adsorption of Cu(II) and Pb(II) ions on SER surface, the thermogravimetric analysis of the samples was performed (see Fig. S4c–e). The herba (aboveground part of the plant) of *Thymus serpyllum* L. (SER) consists of different components with different thermal stability. As can be seen, there are three exothermic peaks at 90 °C, 324 °C and 480 °C on the thermogram of native SER plant. The mass loss at 90 °C includes the loss of moisture present in the sample (~ 10.6 wt%). Next, fast weight losses at 324 °C (~ 53.1 wt%). and 480 °C (~ 33.5 wt%) were detected. The accumulative mass loss at 324 °C was significantly higher than that at 480 °C. These two processes can be considered as the decomposition of the chemical groups on the SER surface. According to [60], the first exothermic peak is related to the decomposition of the carboxylic groups and the second peak can be assigned to the decomposition of the N-containing chemical moieties. The residual mass after the complete burning of the SER plant was near 0.8 wt%, which confirms that the plant consists of organic components.

In the case of the SER sample loaded with Cu(II) ions (Cu-SER sample), the peaks that are related to the carboxylic and N-containing surface groups are shifted on the thermogravimetric diagram. The DTA peak related to carboxylic surface groups is shifted to 310 °C and the peak associated with N-containing groups to 413 °C. This indicates the formation of chemical bonds between the Cu(II) ions and these moieties. Moreover, the additional small endothermic peak at 1009 °C was detected. It can be suggested that this peak corresponds to the Cu(II)  → Cu(I) reduction process or melting of metallic copper [61]. The residual mass after burning was near 5.9 wt. %, which is significantly higher than that of native plant and confirms the successful removal of copper by SER. A similar situation was observed for Pb-SER sample (SER sample loaded with Pb(II) ions). The peaks that are assigned to the carboxylic and N-containing surface groups are shifted on the thermogravimetric diagram: for COOH groups from 324 to 350 °C and for N-containing groups from 480 to 439 °C. The mass of unburned residue was ~ 11 wt%, which was much higher than that obtained after complete burning of the raw SER plant adsorbent and twice as high as that after the adsorption of Cu.

To shed more light on the functional groups potentially involved in the adsorption process, the FT-IR spectra of native SER adsorbent and the Cu-SER and Pb-SER samples were recorded (see Fig. 3g). It is interesting to note that native SER is rich in groups of different nature, which, in our case, is beneficial for the removal of copper(II) and lead(II) ions. The detailed analysis showed that the band corresponding to stretching vibrations of hydroxyl surface groups in native SER plant (broad strong peak ν(–OH)_bonded_ at 3322 cm^−1^) is shifted to ν(–OH)_bonded_ at 3290 cm^−1^ and 3274 cm^−1^ in Pb-SER and Cu-SER samples, respectively, which confirms their participation in the adsorption of the studied metal ions. The surface carboxylic groups possess their stretching vibrations at ν(–C=O) = 1730 cm^−1^ and ν(–C–O) = 1510 cm^−1^ in bare SER plant adsorbent. A shift to ν(–C=O) = 1735 cm^−1^ and no shift for ν(–C–O) = 1508 cm^−1^ in the Pb-SER sample confirm the participation of surface carboxylic groups in the elimination of Pb(II) ions. Similar observations were described in [62], where carboxylic groups were involved in elimination of lead(II) ions using cucumber peels. However, there was no shift in the stretching vibrations of ν(–C=O) and ν(–C–O) for the Cu-SER sample, which demonstrates the absence of interactions between these moieties and the Cu(II) ions. In the case of N-containing surface groups, the large shift in bending vibrations of amino groups (δ(NH_2_) = 1600 cm^−1^, 1629 cm^−1^ and 1614 cm^−1^ for SER adsorbent, Pb-SER and Cu-SER samples, respectively) can be observed. These data are in accordance with the results in [63] and confirm the participation of amino groups in the adsorption process of both metal ions. The bending vibrations of hydroxyl surface groups in the native SER adsorbent δ(C–OH) at 1024 cm^−1^ are related to plant polysaccharide components and are shifted during the adsorption: for Pb-SER sample to δ(C–OH) = 1014 cm^−1^ and for Cu-SER sample to δ(C–OH) = 1016 cm^−1^. Therefore, this verifies the interactions between the hydroxyl moieties of plant components and the Pb(II) and Cu(II) ions. To sum up the observations from FT-IR, the interactions of carboxylic, hydroxyl and amino surface groups play an important role in the adsorptive removal of targeted heavy metals [64].

To investigate the surface chemistry of the pure SER and the corresponding metal-laden samples after the adsorption, XPS method was applied. Namely, the XPS spectra of C 1*s*, O 1*s*, Pb 4*f* and Cu 2*p* were recorded (the last two spectra are provided in Fig. 3h, i, and the rest in Fig. S5) and the changes of binding energies of target elements are shown in Table S2.

The bare SER plant showed the presence of impurities such as fluorine and silicon, which were washed out after the adsorption experiments. Despite the presence of the mentioned impurities, the SER biosorbent plant revealed its organic nature by the characteristic C 1*s* and O 1*s* peaks.

The core-level spectra decomposition of the C 1*s* spectra for pure SER plant was fitted in four main peaks positioned at 284.7 eV, 286.4 eV, 287.9 eV, and 288.9 eV, which are generally assigned to C–C, organic –C–O–, –C=O, and –C–F/COO species, respectively [65]. Similarly, C 1*s* spectra of the SER biosorbent after the Cu(II) and Pb(II) ions adsorption were decomposed into four peaks, with binding energies close to the ones detected for the pure plant. Interestingly, the peak attributed to the –C–O– group increased its intensity at the binding energy of 286.2 eV after the adsorption.

The decomposition of the O 1*s* spectrum suggested that for pure SER biosorbent, the presence of oxygen is only attributed to organic C–O or C=O compounds (binding energy, BE = 532.5 eV) [65, 66]. After the adsorption, the O 1*s* core-level spectra of both Cu-SER and Pb-SER samples were broadened by lateral bumps with deconvoluted peaks around 531.2 eV and 533.7 eV, which are assigned to metal carbonates or oxides and alcohol groups, respectively [66].

The analysis of the Pb 4*f* (Fig. 3i) spectrum revealed the presence of the characteristic doublet with the 4*f*_7/2_ and 4*f*_5/2_ spin–orbital levels distanced by 4.8 eV, suggesting the presence of Pb(II) species, which, after analysing the O 1*s* peak, can be connected to oxidized compounds such as Pb(OH)_2_ or PbO [67, 68]. However, it is difficult to determine the nature of the adsorbed lead substances, as almost all oxygen-containing lead compounds have a peak in this BE range [69].

In the case of Cu adsorption, the characteristic doublet of the Cu 2*p* core-level spectrum (Fig. 3h) presented the spin–orbital levels positioned at 933.3 eV and 953.2 eV, attributed to the Cu 2*p*_3/2_ and Cu 2*p*_1/2_ spin orbitals with a ΔBE of 19.9 eV. The presence of weak intensity satellite features between 938 and 946 eV suggests the presence of Cu(I) species, such as Cu_2_O, rather than Cu(II)-containing compounds [70–72].

**Fig. 4:**
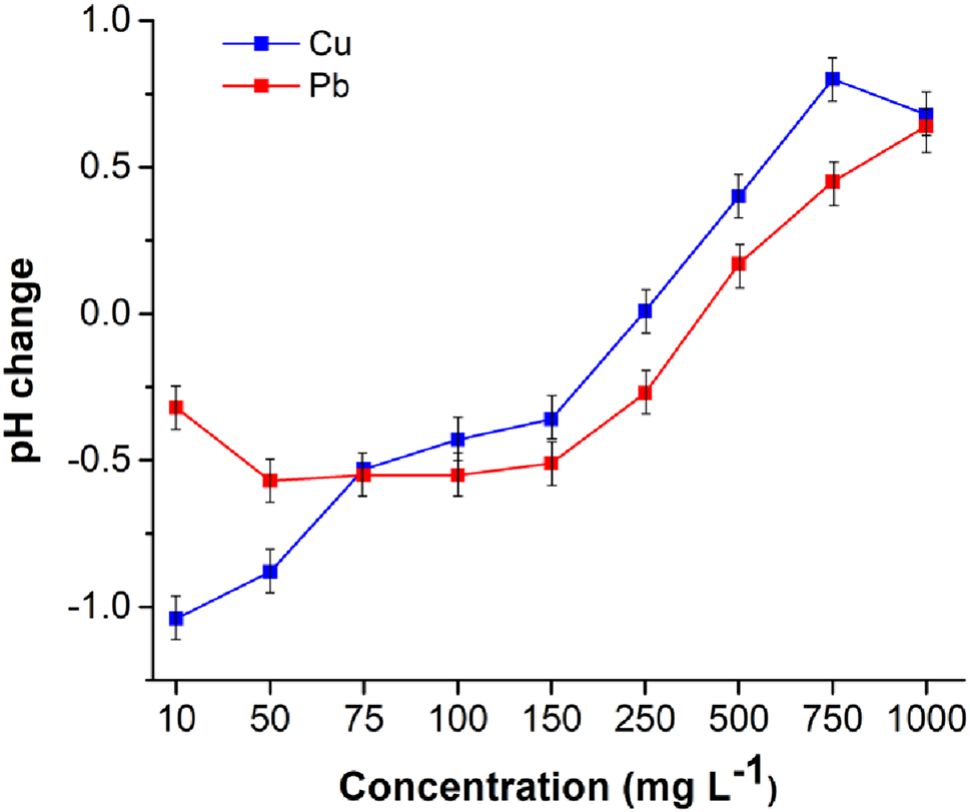
The changes of pH during the adsorption of Cu(II) and Pb(II) ions

The changes in pH that occurred at each point of Cu(II) and Pb(II) adsorption isotherms are illustrated in Fig. 4. The calculation was done by subtracting the final pH after the adsorption from the pH of the starting solution at the beginning of the adsorption (pH_s_–pH_f_). As demonstrated, both curves have negative and positive ranges. At the beginning of the adsorption isotherms (namely, at low concentrations of target ions), the changes of pH are negative, meaning that pH_s_ is lower than pH_f_. This can be explained by the release of alkali and alkaline Earth metals (see Table S3) that increased the pH. This release is related to the ion exchange mechanism of the total adsorption process [73]. Other parts of the curves showed the positive changes of pH, implying that pH_s_ is higher than pH_f_. This can be interpreted as the release of additional hydrogen cations from the reactive carboxylic, amino and hydroxyl groups on the SER surface. It can be concluded that at higher Cu(II) and Pb(II) ions concentrations, complexation and chelation are involved in the adsorption. Therefore, the adsorption of target metals consists of the ion exchange at the lower Cu(II) and Pb(II) ions concentrations and the complexation and chelation at the higher contaminants’ concentrations.

The measurements of ζ-potential for the bare SER plant and the samples Cu-SER and Pb-SER after adsorption showed the decrease of the negative charge after the adsorption processes: namely, the values changed from − 25.4 eV for pure SER sample to − 18.1 eV detected for both the Cu-SER and Pb-SER samples. This can be explained by the binding of negatively charged surface groups (mainly carboxylic) with metal ions.

### Antibacterial activity of the heavy metal-laden adsorbents

**Fig. 5:**
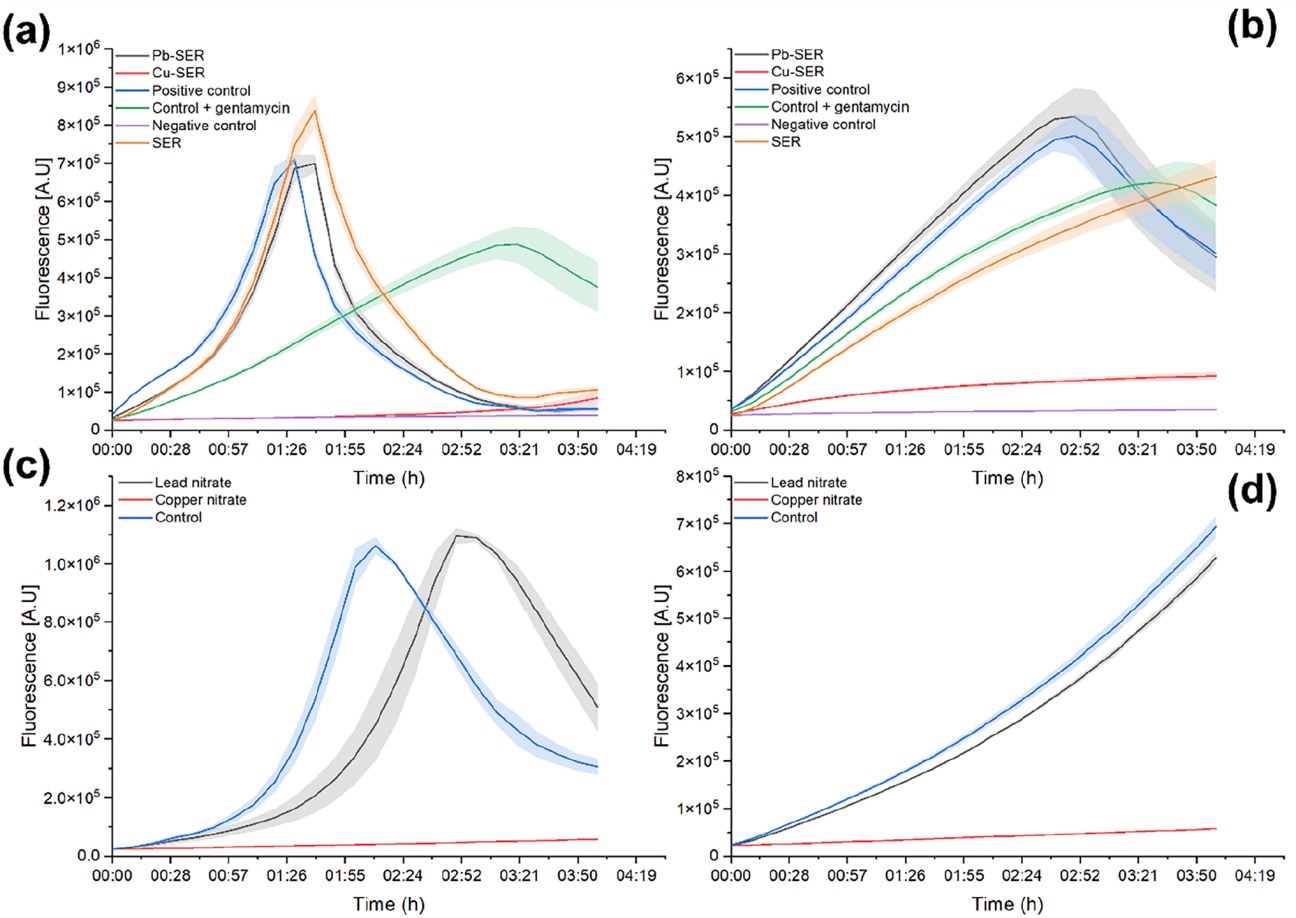
Respiration of **a, c** *E. coli* and **b**, **d** *S. aureus* bacterial cultures after 4-h incubation with pure plant (SER), Cu-laden SER, Pb-laden SER (**a**, **b**) and the solutions of corresponding metal nitrates (**c**, **d**)

To demonstrate the potential re-usability of the Cu-SER and Pb-SER adsorbents, we investigated their antibacterial activity using respiration studies on *Escherichia coli* (*E. coli*) and *Staphylococcus aureus* (*S. aureus*) bacteria and the results are depicted in Fig. 5. Out of the three studied samples (also pure SER plant was investigated), the Cu-SER sample was highly toxic to both bacteria and inhibited their respiration for the duration of the test. The inhibition was more potent than that inflicted by the antibiotic in the selected concentration. Slightly better activity was observed against *E. coli*. On the other hand, the Pb-SER sample did not cause significant changes in the respiration profile. What could be observed was a slight shift in reaching the maximum fluorescence in the case of *E. coli*. For *S. aureus*, the Pb-SER material was not only neutral in the given concentration, but also potentially favourable to its growth. Thus, albeit the adsorption process was less pronounced in the case of Cu(II) ions compared to Pb(II) ions, their presence was satisfactory to cause the antibacterial action. In general, copper-based materials exhibit interesting antibacterial properties [74]. The post-adsorption products containing Cu were tested for the antibacterial action also in two other reports. In [31], no antibacterial properties (evaluated by the MTT assay) against *E. coli* were evidenced for the copper-loaded algae [31]. In [30], the Cu-chitosan/nano-Al_2_O_3_ composite (initially used for As(III) adsorption) exhibited strong antibacterial action (assessed by minimum inhibitory concentration assay) against *E. coli*, whereas the one against *S. aureus* was significantly weaker. This is in accordance with our results. The similarity is also in the simple fact that the presence of Cu(II) ions led to the improvement in the antibacterial action.

For comparison, also pure solutions of copper and lead nitrate in the same concentration, as was calculated according to the maximum sorption capacity values obtained during adsorption tests for the metal-laden adsorbents, were tested for antibacterial action (Fig. 5c, d). Namely, copper nitrate trihydrate of concentration 0.14 mg mL^−1^ (corresponding to adsorption capacity 12.66 mg g^−1^) and lead nitrate of concentration 0.17 mg mL^−1^ (corresponding to 53.13 mg g^−1^) were used. Both solutions showed a potential to inhibit respiration in both studied bacterial populations. Similar to the metal-laden adsorbents, the antibacterial effect was much stronger for copper nitrate than for lead nitrate. When comparing the results of metal-laden adsorbents with that of pure solutions, the results were slightly better in the latter case, most probably due to the better mobility of the ions due to liquid environment. This can be also demonstrated on the example of lead, where the Pb-SER sample showed negligible inhibition (*E. coli*) or even stimulating the growth of bacteria (*S. aureus*), whereas in the case of lead ions-bearing solution, a significant and a slight inhibition was evidenced in the case of *E. coli* and *S. aureus*, respectively. However, the difference in antibacterial action between the solutions and metal-laden adsorbents is not large, and taking into account that with the same material both water purification and subsequent antibacterial action can be achieved, the proposed methodology seems to be beneficial.

The results for the pure SER plant were the opposite in comparison with that of metal-laden adsorbents and metal ions solutions. While *E. coli* may even be stimulated by the presence of SER, the respiration of *S. aureus* was inhibited. A significant antibacterial activity of the essential oil made from *Thymus serpyllum* L. against *S. aureus* was evidenced also in [75]. The difference might be connected with differences in the biology of selected bacteria, as the former one is Gram negative, e.g. having thinner cell wall and outer membrane [76], whereas the latter is Gram positive with thicker and more rigid cell [77]. These and other differences (such as the ones in stress response mechanisms) differentiate the action of the species present in the plant.

## Conclusions

The proposed research work showed the native eco-friendly plant *Thymus serpyllum* L. (wild thyme) to be a promising biosorbent of Cu(II) and Pb(II) ions. Good adsorption ability was evidenced despite the plant was used in its native state (no biochar was produced). The combined adsorption in the binary metal system showed very high removal efficiency of Pb(II) ions (90.1%) and only moderate one of Cu(II) ions (61.5%). The adsorption kinetics was found to be very rapid and followed the pseudo-second-order model with the correlation coefficients 0.9997 and 0.9961 for Cu(II) and Pb(II) ions, respectively. According to the isotherm modelling, the Langmuir model described the adsorption of Cu(II) ions better, which implied that the adsorption of Cu(II) ions was limited to one molecular layer. The adsorption of Pb(II) ions was better defined by the Freundlich isotherm model, which suggested that the adsorption occurred on the heterogeneous surface of the biosorbent. The highest experimentally obtained adsorption capacity was 12.66 mg g^−1^ after 10 min of adsorption at 16 g L^−1^ adsorbent dosage for Cu(II) ions and 53.13 mg g^−1^ after 30 min of adsorption at 12 g L^−1^ adsorbent dosage for Pb(II) ions. The TGA and FT-IR results revealed that the hydroxyl and amino surface groups played an important role during the adsorption of both metal ions and in Pb(II) ions adsorption, also the carboxylic groups were involved. The XPS analysis of the samples after the adsorption revealed that Cu(II) ions were adsorbed in the form of Cu(I) species (most probably Cu_2_O) and Pb(II) ions as oxidized compounds such as Pb(OH)_2_ or PbO. The possible adsorption mechanism consists of ion exchange at lower Cu(II) and Pb(II) concentrations and complexation and chelation at higher concentrations of the targeted heavy metal ions. The copper-laden adsorbent was identified as a promising antibacterial agent by respiration study. Its activity was higher than that of pure plant and it was efficient against both *E. coli* and *S. aureus*. The efficient embedding of copper ions as a result of bisosorption process within the biological matrix of the plant seems to be a key process to trigger the antibacterial action. As this effect was not observed in the case of pure plant, it has to be triggered by the presence of the ions. Thus, the investigated biosorbent could be re-used as an antibacterial agent in the next step, which clearly shows the sustainable character of the proposed approach.

### Supplementary Information

Below is the link to the electronic supplementary material.

Supplementary file1 (DOCX 1662 kb)

## Data Availability

The datasets generated during and/or analysed during the current study are not publicly available because they have not been uploaded to any public repository, but are available from the corresponding author on reasonable request.
