# Supplementary material for: Triggering antibacterial activity of a common plant by biosorption of selected heavy metals
Source: J Biol Inorg Chem. 2024 Apr 8;29(2):201–16. doi: 10.1007/s00775-024-02045-1 (PMC11098919; doi:10.1007/s00775-024-02045-1)
Supplement: Supplementary file 1 — Supplementary file1 (DOCX 1662 kb) [file 775_2024_2045_MOESM1_ESM.docx]

**Supplementary information**

For the paper

**Triggering antibacterial activity of a common plant by biosorption of selected heavy metals**

**Mária Kováčová ^1^, Halyna Yankovych ^1^, Adrian Augustyniak ^2,3,4^, Mariano Casas Luna ^5,6^, Michaela Remešová ^5^, Lenka Findoráková ^1^, Martin Stahorský ^1^, Ladislav Čelko ^5^, Matej Baláž ^1,*^**

^1^ Institute of Geotechnics, Slovak Academy of Sciences, Watsonova 45, 040 01 Košice, Slovakia

^2^ Chair of Building Materials and Construction Chemistry, Technische Universität Berlin, Gustav-Meyer-Allee 25, 13355 Berlin, Germany

^3^ Faculty of Chemical Technology and Engineering, The West Pomeranian University of Technology in Szczecin, Piastów Avenue 42, 71 065 Szczecin, Poland

^4^ Institute of Biology, University of Szczecin, ul. Wąska 13, Szczecin, 71-415, Poland

^5^ Central European Institute of Technology, Brno University of Technology, Purkyňova 656/123, 612 00 Brno, Czech Republic

^6^ Faculty of Mathematics and Physics, Charles University, Ke Karlovu 3, 121 16 Praha 2, Czech Republic

**^*^** corresponding author: **balazm@saske.sk**


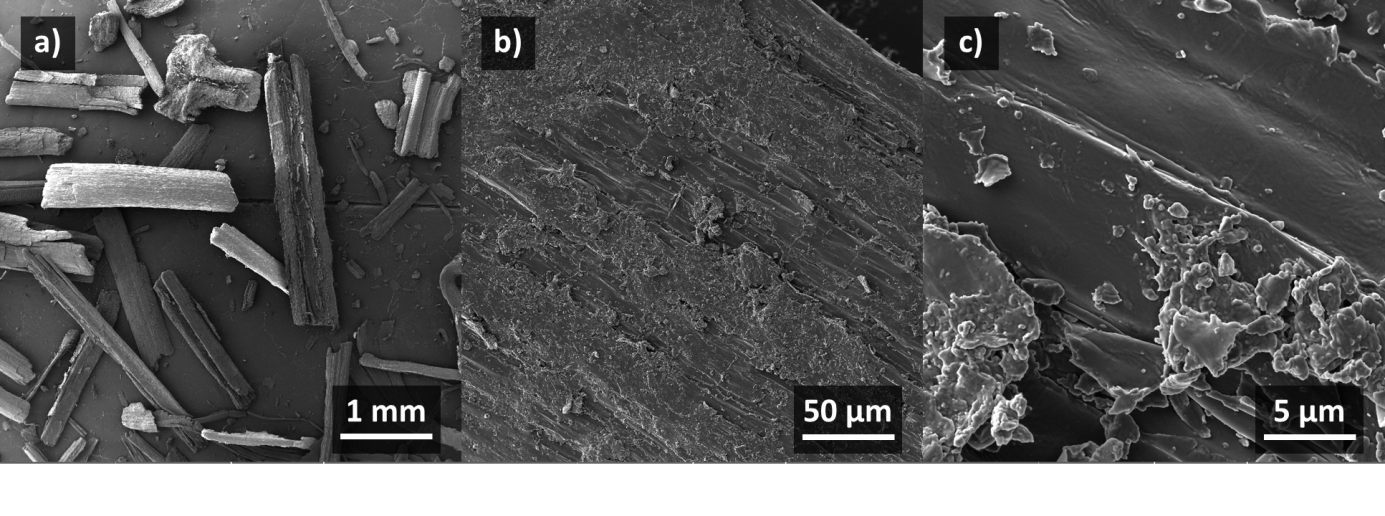


Figure S1 SEM micrographs of the initial Thymus serpyllum L. (SER) plant.


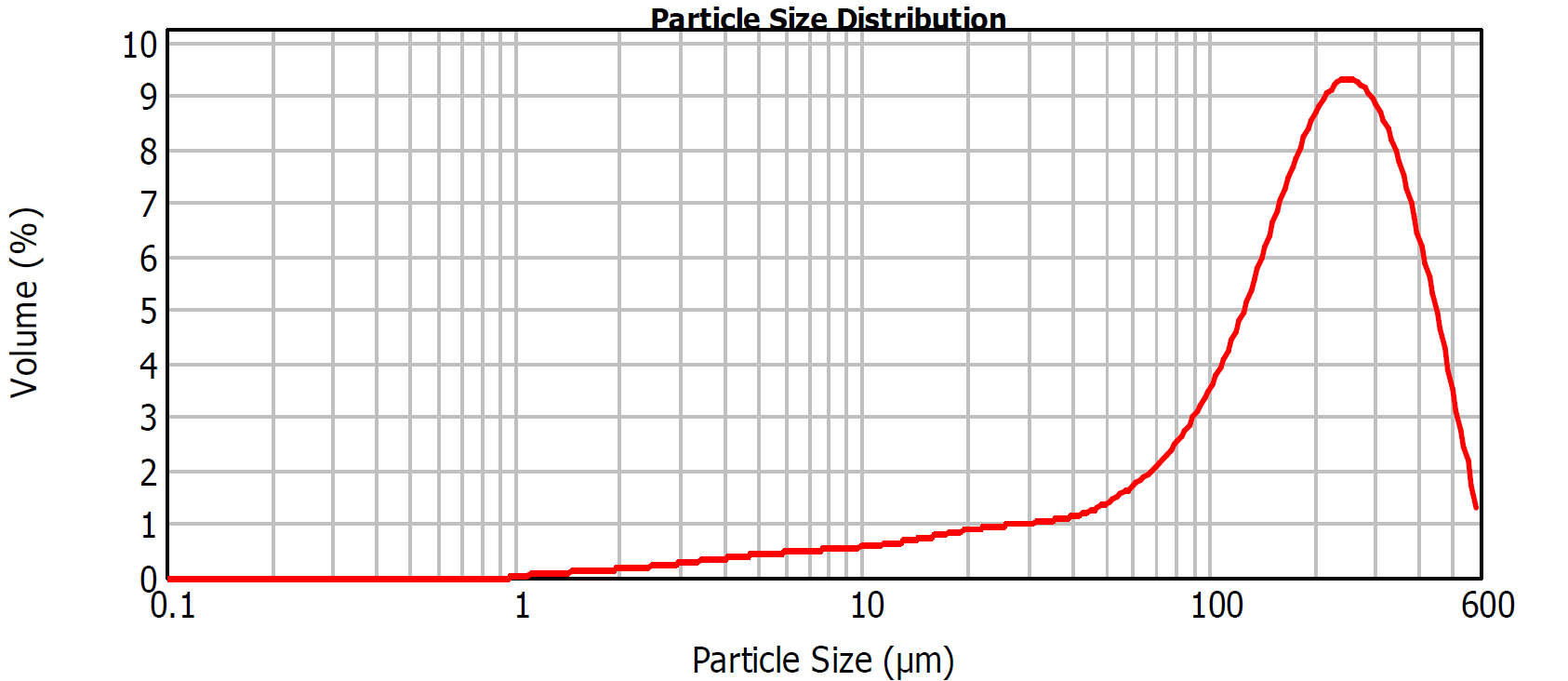


Figure S2 Particle size distribution of the initial Thymus serpyllum L. (SER) plant. The d10, d50 and d90 values were 29.413 µm, 195.993 µm and 400.395 µm, respectively

Table S1 The results of preliminary experiment with model solution

| **Sample** | **Concentration of element (mg L^-1^)** | | | | | | |
| --- | --- | --- | --- | --- | --- | --- | --- |
|  | **Fe** | **Cu** | **Zn** | **Co** | **Cd** | **Al** | **Pb** |
| **Model solution** | 18.9 | 20.1 | 18.2 | 20.4 | 20.1 | 19.7 | 22.1 |
| ***Thymus serpyllum* L.** | 5 | 11.6 | 14.3 | 16.4 | 16.2 | 14.7 | 7.5 |

Figure S3a shows the adsorption capacity (q_t_) values for the adsorption of Cu(II) ions at three different concentrations. The obtained results were the most consistent at c = 200 mg L^-1^. The q_t_ value at c = 1000 mg.L^-1^ after 5 minutes was the highest of all samples (q_t_ = 42.25 mg g^-1^), however, the rest of the results obtained at this concentration were not representative, most probably due to the additional dilution which created the error. The adsorption capacity was below 8 mg g^-1^ for all samples at c = 20 mg L^-1^. The highest q_t_ value for c = 200 mg L^-1^ was acquired after 10 minutes of adsorption (q_t_ = 28.6 mg g^-1^).


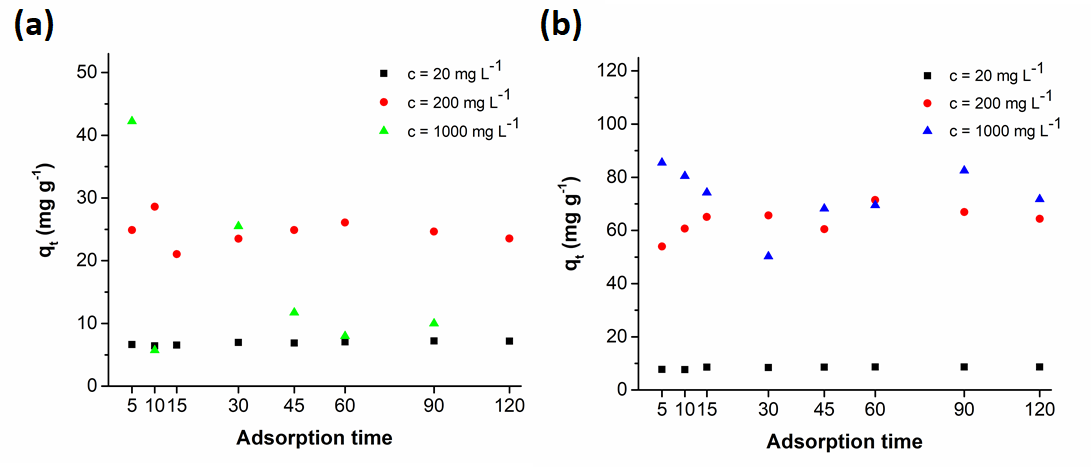


Figure S3 Adsorption of Cu(II) (a) and Pb (II) ions at three different concentrations

Fig. S3b displays the q_t_ values for the adsorption of Pb(II) ions at three different concentrations. We obtained the lowest q_t_ values at c = 20 mg L^-1^ (q_t_ < 9 mg g^-1^) which was quite distinct from the other two concentrations. During first 15 minutes of the adsorption, the adsorption capacity was better at c = 1000 mg L^-1^ but after 30 minutes, better q_t_ value (q_t_ = 65.65 mg g^-1^) was achieved at c = 200 mg L^-1^. The adsorption capacity for the samples after 45 minutes at concentrations 200 and 1000 mg L^-1^ were not very different from the sample at c = 200 mg L^-1^ after 30 minutes of adsorption. The slightly better adsorption capacities were achieved at c = 200 mg L^-1^ after 60 minutes (q_t_ = 71.45 mg g^-1^) and at c = 1000 mg L^-1^ after 90 and 120 minutes of contact time and the adsorption capacities were 82.5 mg g^-1^ and 71.75 mg g^-1^, respectively.


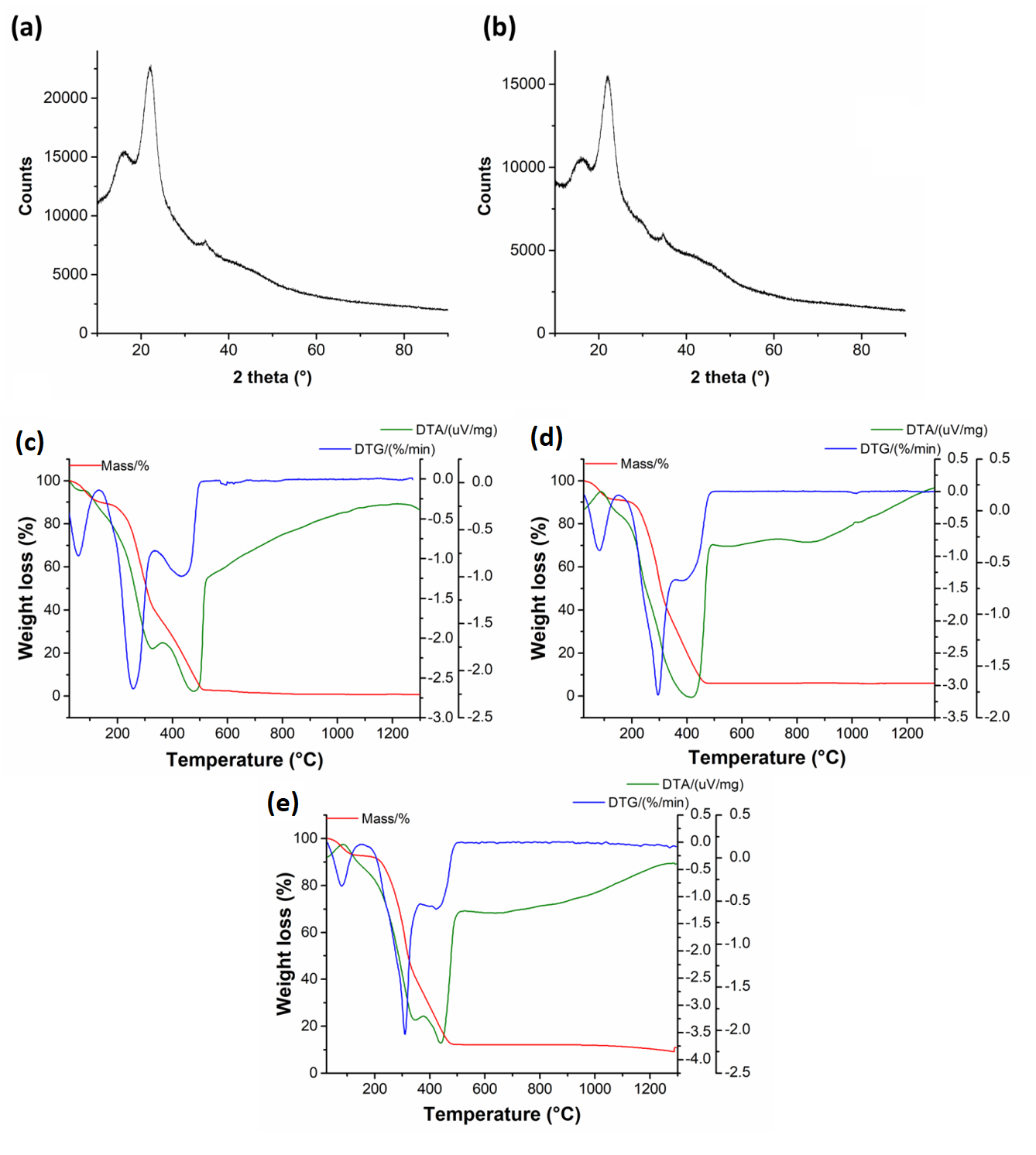


Figure S4 Characterization of metal-laden adsorbents: X-ray diffractograms of (a) Cu-SER and (b) Pb-SER samples; TG/DTG and DTA curves of (c) native *Thymus serpyllum* biosorbent and (d) Cu-SER and (e) Pb-SER samples.


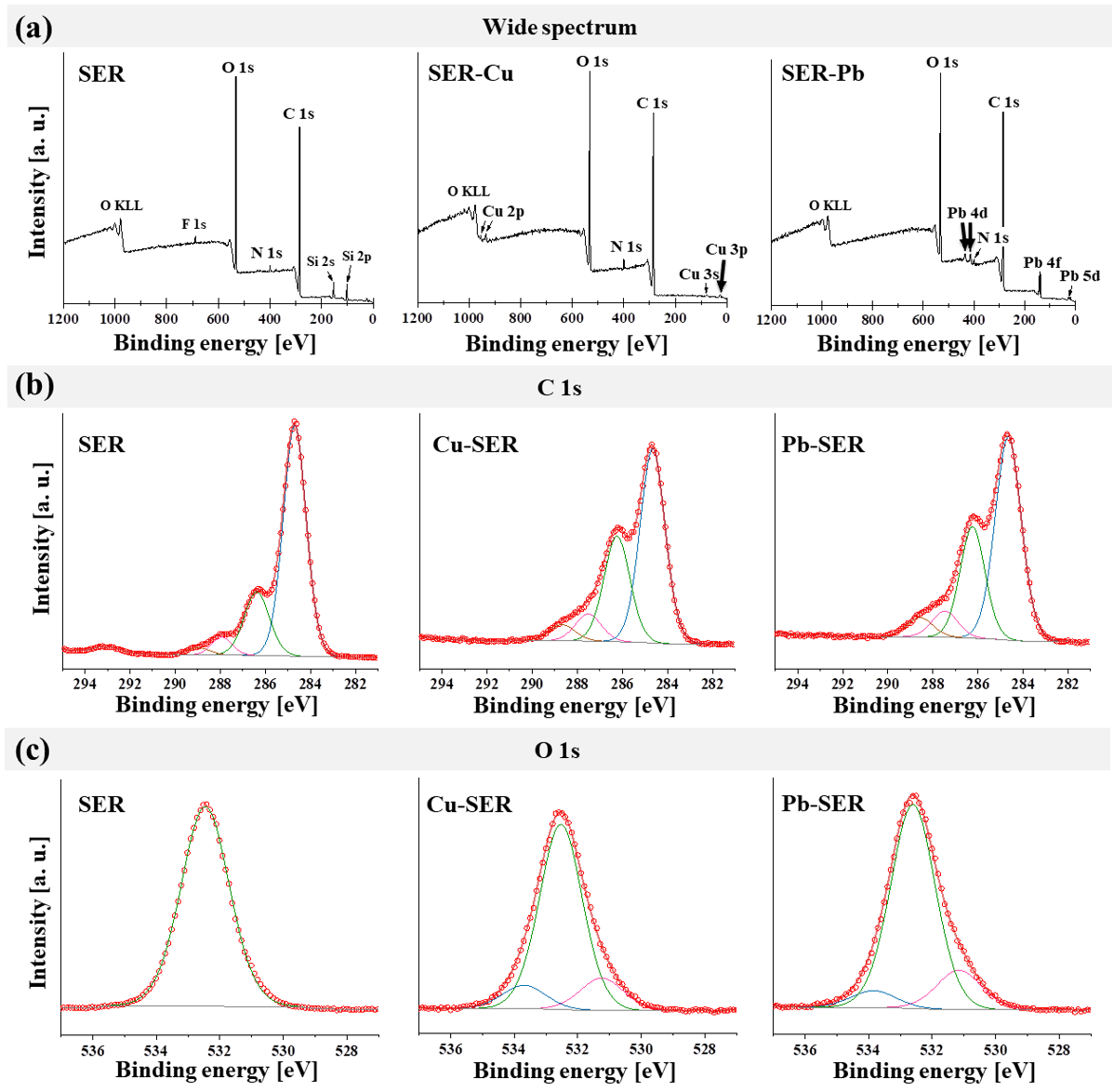


Figure S5 XPS spectra of pure SER biosorbent before and after Cu and Pb adsorption: a) survey spectra; b) high-resolution spectra and deconvolution of C 1s peak; c) high-resolution spectra and deconvolution of O 1s peak.

**Table S2** The binding energies of C1s, O1s, N1s, Pb4f and Cu2p before and after adsorption

| **Element** | **SER** | **Pb-SER** | **Cu-SER** | **Assignment** |
| --- | --- | --- | --- | --- |
| **O 1s** | - | 531.2 eV | 531.4 eV | Metal carbonates, metal oxides, metal hydroxides |
|  | 532.5 eV | 532.6 eV | 532.5 eV | Organic C-O-, C=O, |
| **N 1s** | 399.9 eV | 399.8 eV | 399.9 eV | -NH-; -NH_2_ |
| **C 1s** | 284.7 eV | 284.7 eV | 284.7 eV | Graphite, C-C, -CH- |
|  | 286.4 eV | 286.2 eV | 286.2 eV | -C-O-C-, -C-OH, ether -C-O |
|  | 287.9 eV | 287.5 eV | 287.5 eV | -C=O |
|  | 288.9 eV | 288.6 eV | 288.2 | -C-F, O=C-OH |
| **Pb 4f** | - | 138.9 eV 4f_7/2_  143.7 eV 4f_5/2_  Δ=4.8 eV | - | Pb(OH)_2_, PbCO_3_ |
| **Cu 2p** | - | - | 933.3 eV 2p_3/2_  953.2 eV 2p_1/2_  Δ=19.9 eV | CuO |

Table S3 The release of alkali- Na(I), K(I) and alkaline earth- Ca(II), Mg(II) metals into the aqueous solution of pure *Thymus serpyllum* L. plant

|  | **Concentration of element (mEq g^−1^)** | | | | |
| --- | --- | --- | --- | --- | --- |
| **Sample** | **Na** | **K** | **Ca** | **Mg** | **∑** |
| **Cu concentration (mg L^-1^)** |  |  |  |  |  |
| 10 | 0.10 | 12.12 | 2.01 | 3.44 | 17.67 |
| 50 | 0.12 | 11.26 | 2.02 | 3.35 | 16.75 |
| 75 | 0.12 | 11.69 | 2.42 | 3.82 | 18.05 |
| 100 | 0.10 | 12.12 | 3.05 | 4.29 | 19.56 |
| 150 | 0.15 | 11.41 | 4.26 | 4.51 | 20.34 |
| 250 | 0.20 | 13.07 | 8.33 | 6.42 | 28.02 |
| 500 | 0.12 | 12.25 | 8.07 | 6.23 | 26.68 |
| 750 | 0.14 | 12.67 | 8.44 | 6.60 | 27.84 |
| 1000 | 0.14 | 12.82 | 9.43 | 7.15 | 29.54 |
| ***Thymus serpyllum* L.** | 0.11 | 11.02 | 1.63 | 2.86 | 15.62 |
| **Pb concentration (mg L^-1^)** |  |  |  |  |  |
| 10 | 0.2 | 9.7 | 1.8 | 3.0 | 14.7 |
| 50 | 0.1 | 11.0 | 2.1 | 3.5 | 16.8 |
| 75 | 0.1 | 9.7 | 2.1 | 3.3 | 15.2 |
| 100 | 0.1 | 10.7 | 2.8 | 4.5 | 18.0 |
| 150 | 0.1 | 11.1 | 3.2 | 4.4 | 18.8 |
| 250 | 0.1 | 10.7 | 4.2 | 3.6 | 18.7 |
| 500 | 0.2 | 10.5 | 5.4 | 4.3 | 20.4 |
| 750 | 0.3 | 17.9 | 9.3 | 8.1 | 35.6 |
| 1000 | 0.1 | 10.4 | 7.2 | 4.8 | 22.5 |
| ***Thymus serpyllum* L.** | 0.1 | 10.4 | 2.0 | 3.4 | 16.0 |
